# A way forward for teaching and learning of Physiology: Students’ perception of the effectiveness of teaching methodologies

**DOI:** 10.12669/pjms.326.10120

**Published:** 2016

**Authors:** Rabiya Rehan, Khalid Ahmed, Hira Khan, Rehana Rehman

**Affiliations:** 1 Dr. Rabiya Rehan, MBBS. Senior Lecturer, Department of Physiology, Bahria University Medical and Dental College, Karachi, Pakistan; 2 Dr. Khalid Ahmed, MBBS. Senior Instructor, Department of Biological and Biomedical Sciences, Faculty of Health Sciences, Agha Khan University, Karachi, Pakistan; 3 Hira Khan, MS. Research Scholar, National University of Sciences and Technology (NUST), Karachi, Pakistan; 4 Dr. Rehana Rehman, Ph.D. Physiology. Assistant Professor, Department of Biological & Biomedical sciences Aga Khan University, Karachi, Pakistan

**Keywords:** Students’, perceptions, Case-based sessions, Interactive lectures, Physiology teaching and learning

## Abstract

**Objective:**

To compare the perception of medical students on the usefulness of the interactive lectures, case-based lectures, and structured interactive sessions (SIS) in teaching and learning of Physiology.

**Methods:**

A cross-sectional study was carried out from January to December 2012 at Bahria University Medical & Dental College, Karachi, which had qualitative and quantitative aspects, assessed by self- reported questionnaire and focused group discussion (FGD). The questionnaire was distributed to 100 medical students after completion of first year of teaching of MBBS Physiology. The data was analyzed using SPSS version 15. Differences were considered significant at p-values <0.05 after application of Friedman test. Responses of FGD were analyzed.

**Results:**

All the teaching methodologies helped in understanding of precise learning objectives. The comprehension of structure and functions with understanding of difficult concepts was made best possible by SIS (p=0.04, p<0.01). SIS enabled adult learning, self-directed learning, peer learning and critical reasoning more than the other teaching strategies (p< 0.01).

**Conclusion:**

SIS involved students who used reasoning skills and power of discussion in a group to comprehend difficult concepts for better understanding of Physiology as compared to interactive and case-based lectures.

## INTRODUCTION

Physiology is bedrock of undergraduate medical curriculum. The preference for a particular mode of content delivery has been extensively investigated by medical teachers to convey knowledge in a logical, strategic, cohesive and chronological manner to the students.^1^ The times are changing and more emphasis has been placed now on the development of critical thinking skills in contrast to emphasis on the systems-based didactic lectures.^2^ Physiology has been recognized as a challenging discipline for medical students to comprehend, integrate and apply in clinical sciences. Moreover, students exclusively face difficulty in understanding core physiological concepts in the context of disease processes and may require help from physiologists.^3^,^4^ Medical educationists are thus looking at ways to achieve effective vertical and horizontal integration in the discipline of Physiology.^5^

Regular assessment of the medical curriculum is the need of time to further improve the living document of curriculum by cultivating and introducing various new teaching and learning approaches. Multiple teaching and learning approaches are used for teaching of Physiology like interactive lectures, (IL) structured interactive sessions (SIS), case-based lectures (CBL) and problem-based learning (PBL) techniques.^6^ It is important for Physiology faculty to inform themselves about the discipline, various teaching and learning strategies and how effective those methods are to achieve the goal of the real student learning.^7^

Didactic lectures have evolved into interactive IL along with delivery of core conceptual understanding of physiological mechanisms. When lectures are properly structured, teachers are in a better position to keep students engaged and motivated in the classroom. Nevertheless, as an effective teaching and learning technique, the case-based instruction and discussion surpasses the IL in terms of actual student learning experience.^8^ This supports the assertion that students feel motivated and empowered when they are helped to actively engage in self-directed learning exercises.^9^

Small Group Tutorials smoothly develop discussion on prior content knowledge with perceptions of ideas and facts from text books or lectures. Researchers have demonstrated that they enhance individual attention of teacher to facilitate equal participation by all students, which improves the students’ interest as well as performances.^10^ These sessions have been receiving consistent positive feedback in terms of understanding the concepts of Physiology. SIS encourages increased transaction of knowledge between teachers and students with their active participation, motivation and interaction.^11^

Case-based learning (CBL) is a major teaching and learning method.^12^ In case-based discussions, it is aimed to understand theoretical functional aspects of basic sciences and correlating those facts to the clinical signs and symptoms and pathophysiological processes. This method develops and improves the complex-problem solving skills of the students. The disease processes deals with deeper understanding of physiological and pathological aspects. In this scenario, the emphasis is not physiological understanding alone but as integration of the pathological underpinnings of the physiological concepts.^13^

As a result of incorporation of PBL in the curriculum of Bahria University Medical & Dental College,^14^ the decision makers wanted to explore perception of students in terms of IL, CBL and SIS so as to decide and assign their due weightage in the curriculum. We aimed to explore the perceptions of medical students about the usefulness of IL, CBL and SIS as major teaching and learning methodologies in Physiology.

## METHODS

In the academic year, twenty eight IL were taken in large class format of 100 students by designated senior faculty members (Professors and Associates) in the scheduled time frame. The CBLs (12) on specific topics were taken in large class formats and the clinical scenarios were prepared by group of physiologists and clinicians. The SIS (25) were conducted after the interactive and case based lectures on given objectives by lecturers and senior lecturers. The tutorial objectives were displayed on the departmental notice boards ahead of time and students were directed to come prepared with the content. These sessions were conducted in a batch of 33 students each.

A cross-sectional study was carried out from January to December 2012 at Bahria University Medical & Dental College, Karachi, which had qualitative and quantitative aspects, assessed by self- reported questionnaire and focused group discussion (FGDs). The questionnaire was distributed to 100 medical students after completion of first year of teaching of MBBS Physiology.

### Quantitative Analysis

The questionnaire had two components; (Annexure Ia) was meant to analyze perception of students in terms of learning of content, concepts and objectives of Physiology and integration of acquired knowledge with other subjects. The responses were graded as poor, satisfactory, good, very good and excellent from 1 to 5. The skills and capabilities acquired with use of IL, CBL and SIS were evaluated by second component of the questionnaire (Annexure I b). The answers were assessed on the scale not at all, to some extent, to a great extent, to a complete extent from 0 till 3. The reliability of the questionnaire was established by Cronbach’s Alpha (81%) which revealed good uniformity in the responses received from students. All the students who had attended 21/28 IL, 19/25 SIS and 9/12 CBL during two years were recruited in the study. Data was entered and later analyzed by using SPSS version 15. Friedman test was used to test for differences between groups as the dependent variable being measured was ordinal values, significance at at p-values <0.05.

### Qualitative Analysis

For the qualitative analysis, FGDs were conducted in three groups comprising of 10 students selected by simple random selection from A, B and C batches of the class, two weeks after the questionnaire was administered. The interview guide for FGD was developed by the researchers from an iterative literature process. The interview guide was piloted on the students who are not participating in the study. The FGDs were carried out after informed consent in the main conference room for a period of 30 minutes in which they were asked about strength and weaknesses of IL, CBL and SIS with suggestions and recommendations. They were audio recorded; anonymity and confidentiality of students was maintained throughout entire process. The audio recording was transcribed and approved by the students before dissemination. To avoid any bias, FGDs were conducted by faculty not involved in teaching of basic medical Sciences.

## RESULTS

### Quantitative Section

A response rate of 88% was acquired from the students. Results showed that learning objectives were understood by all teaching methodologies (Table-I). The structure and functions was understood better by SIS in comparison to other teaching tools (p=0.04). The recognition of difficult concepts was made the best possible by SIS (p<0.01). The mean scores in Table-II indicate the importance of SIS for adult learning, self-directed learning, peer learning and critical reasoning as compared to IL and CBL (p< 0.01).

**Table-I:** Perception of students in terms of usefulness of teaching methodologies in the subject of Physiology.

Contents	Mean Rank			p-value

	Interactive Lectures	Structured Interactive sessions	Case Base Session	
Understanding of learning objectives	3.98	4.13	3.89	0.153
Association of structure and function	2.09	3.09	2.83	0.04*
Recognition of difficult concepts	3.97	4.30	3.73	<0.01*
Comprehension of pathogenesis	3.91	3.09	3.91	0.39
Recall and assimilation of knowledge	3.07	3.03	2.90	0.35
Achievement of good university grades	3.91	4.03	4.06	0.46

Mean Ranks were acquired with representation of 1 as Poor, 2 as Satisfactory, 3 as Good, 4 as Very Good and, 5 as Excellent. Results were compared by Friedman test

* p <0.05.

**Table-II:** Use of Teaching Methodologies (Lectures, case based lectures and interactive sessions) for development of skills and capabilities.

	Lectures	Structured Interactive sessions	Case Based Lectures	P value
Active learning	1.85	2.10	2.05	0.08
Self-directed learning	1.79	2.12	2.09	<0.01]
Peer learning	1.65	2.20	2.15	<0.01*
Critical reasoning	1.74	2.04	2.22	<0.01*
Presentation skills	1.90	2.21	1.89	<0.01*

Mean rank of response of interactive sessions compared with lectures and case based lectures using Friedman test

* P value considered significant at <0.05

**Table:** Appendix-1a

Analysis of students perception on teaching tools. Rate the usefulness of following teaching/learning methods employed. SCALE 1= Poor, 2= Satisfactory, 3= Good, 4=Very Good, 5=Excellent						

Objective	Teaching Methodology	Poor	Satisfactory	Good	Very Good	Excellent
Active learning	Case Based Lecture					
	Interactive Lecture					
	Small group Interactive session					
Self-directed learning	Case Based Lecture					
	Interactive Lecture					
	Small group Interactive session					
Peer learning	Case Based Lecture					
	Interactive Lecture					
	Small group Interactive session					
Critical reasoning	Case Based Lecture					
	Interactive Lecture					
	Small group Interactive session					
Presentation skills	Case Based Lecture					
	Interactive Lecture					
	Small group Interactive session					

### Qualitative Section

Students criticized the subjective variation in the discussions generated by different instructors who were designated to take SIS. One of the students mentioned, “Sometimes the instructor discusses more than what is required as per learning objectives.” Few students proposed that for CBL, live patients could be brought from hospital and if not possible a role-play should be introduced to understand the relevant concepts. “The lecturers use images in the lectures without showing the copy rights/source” was a comment made by one of the student. Students also proposed the need of integrated lectures to be taken as a joint venture by multi-disciplinary facilitators. “We often sleep, or play on our cell phones in the boring lectures” was a very frequent statement made by good number of students. They also recommended that the component of IL to be reduced without any change in SIS and CBL weightage in the curriculum.

## DISCUSSION

Studies have shown that introduction of case-based sessions using problem-based learning techniques may help students to compensate for the inherent weaknesses of systems-based discipline-based traditional medical curricula.^15^ The provision of such interactive teaching and learning sessions not only motivates and actively engages the students, but also increases their involvement in lectures and classrooms.^16^

**Table:** Annexure-1b

Analysis of usefulness of following teaching/ learning methods for development of learning capabilities. Rate the usefulness of following teaching/learning methods employed. SCALE 0= Not at all, 1= To some extent, 2= To a great extent 3= To the full extent					

Objective	Teaching Methodology	Not at all	To some extent	To a great extent	To the full extent
Enabled active learning	Case Based Lecture				
	Interactive Lecture				
	Small group Interactive session				
Motivated selfdirected learning	Case Based Lecture				
	Interactive Lecture				
	Small group Interactive session				
Developed skill of working in group	Case Based Lecture				
	Interactive Lecture				
	Small group Interactive session				
Learned skill of critical reasoning	Case Based Lecture				
	Interactive Lecture				
	Small group Interactive session				
Learned skill of presentation	Case Based Lecture				
	Interactive Lecture				
	Small group Interactive session				

It is said that the interactive lecture-based teaching method would continue to be practiced in foreseen future,^1^ because of its utility in terms of economical way of imparting large chunks of information to the students in a large class.

Didactic lectures are the most commonly used forms of content delivery method that requires a heavy time-commitment of the teacher. It has been found to be of a little benefit to the students in terms of actual learning experience and the limited active engagement with the content, which is contrary to the results published by previous researchers.^17^,^18^

Students recommended enhanced interchange of information with the facilitators which has also been proved to be very effective by other researchers.^16^ This can be explained on the basis of the fact that when the information is taught to them via interactive methods, it is perceived to have retained greater amounts of retention for the longer periods of time. When lectures are properly structured, teachers are in a better position to keep students engaged and motivated in the classroom.

In this study, it was found that actual understanding of the structure and functions of the physiological content was best perceived when they were delivered through SIS. The observation is supported by the observations in which interactive sessions showed marked improvement in students’ learning over traditional model of teaching.^19^

Active learning is often focused on the active involvement of students are considered as the best educational practice in the classrooms.^20^ Students considered SIS to be the most effective method helpful in the understanding of difficult concepts. Numerous studies have shown a positive student feedback regarding more interactive approaches to teaching and learning which helps in understanding of difficult concepts as well.^21^ It has been revealed that interactive educational approaches involve greater student involvement in student learning processes.

It has been shown that the problem-solving skills of the students develop through self-directed learning which enables and enhances critical thinking and reasoning skills for learning of students.^22^ By this mechanism learner takes the initiative and remains responsible for his own learning by setting definite learning goals. This identifies gaps in learning to adopt the strategies that prove instrumental throughout their careers as physicians and educators.^23^ The element of self-directed learning was reinforced by SIS in which students took the responsibility of their learning.

Students’ learning abilities shows immense improvement, while working in the small and large groups in interactive sessions compared to large class formats. As students tend to implement and discuss the knowledge shared to them with rest of the class this lead to efficient comprehension of the material being taught to them. Students reported that they learnt great amount of knowledge by sharing of their ideas and discussing it with their peers which was observed in surveys by other researchers.^24^ Clinical problems have the capacity to convert boring didactic lecturing of the classroom in to healthy active mental activity for students.^25^ In a study, CBL proved to be an effective way to integrate newly acquired information and provided a unique platform to integrate disciplines to understand pathogenesis of the disease and solve complex clinical problems.^13^ This information would help the stakeholders to make informed decisions regarding impending curricular changed.^26^ The suggestions given during FGD can further help in launching innovations in this teaching strategy.

Active learning is capable of increasing student retention of knowledge in any field of information and technology.^27^ Numerous studies conducted in Europe, Canada and America in the medical education found out that students learn and apply their knowledge more effectively when didactic lectures are supplemented with clinical cases and accompanied with interactivity during the classrooms. This study finds that the students showed significant improvement in clinical reasoning with the help of CBL as compared to IL and SIS.

The study is limited in terms of lack of validation of questionnaire, inequality of teaching hours and the differences in comparison of large with small class format. Although the students were informed about teaching evaluation in the beginning of year, and they kept on taking notes in each session, yet was really tough for the students to recall all the sessions and comment on them. It however, is expected to provide a useful platform to undertake further in-depth understanding of how the student perceptions will be shaped to bring change in the curricular outcomes by the decision makers for the actual end-users of the curriculum.

## CONCLUSION

Use of interactive educational approaches helped in understanding of the subject as well as improved the learning capabilities of medical students. SIS involved students who used reasoning skills and power of discussion in a group to comprehend difficult concepts for better understanding of Physiology in relevance to other subjects. The importance of CBL cannot be overlooked in terms of orientation with clinical cases, however improvement in the structure of IL to make it more interactive is what is the demand of the students.
